# Integrated radiogenomics analyses allow for subtype classification and improved outcome prognosis of patients with locally advanced HNSCC

**DOI:** 10.1038/s41598-022-21159-7

**Published:** 2022-10-06

**Authors:** Asier Rabasco Meneghetti, Alex Zwanenburg, Annett Linge, Fabian Lohaus, Marianne Grosser, Gustavo B. Baretton, Goda Kalinauskaite, Ingeborg Tinhofer, Maja Guberina, Martin Stuschke, Panagiotis Balermpas, Jens von der Grün, Ute Ganswindt, Claus Belka, Jan C. Peeken, Stephanie E. Combs, Simon Böke, Daniel Zips, Esther G. C. Troost, Mechthild Krause, Michael Baumann, Steffen Löck

**Affiliations:** 1 OncoRay-National Center for Radiation Research in Oncology, Faculty of Medicine and University Hospital Carl Gustav Carus, Technische Universität Dresden, Helmholtz-Zentrum Dresden-Rossendorf, Fetscherstraße 74, 01307 Dresden, Germany; 2 Institute of Radiooncology-OncoRay, Helmholtz-Zentrum Dresden-Rossendorf, Dresden, Germany; 3 German Cancer Consortium (DKTK), Partner Site Dresden, Germany and German Cancer Research Center (DKFZ), Heidelberg, Germany; 4 National Center for Tumor Diseases (NCT), Partner Site Dresden, Germany: German Cancer Research Center (DKFZ), Heidelberg, Germany; Faculty of Medicine and University Hospital Carl Gustav Carus, Technische Universität Dresden, Dresden, Germany, and Helmholtz Association / Helmholtz-Zentrum Dresden – Rossendorf (HZDR), Dresden, Germany; 5 Department of Radiotherapy and Radiation Oncology, Faculty of Medicine and University Hospital Carl Gustav Carus, Technische Universität Dresden, Dresden, Germany; 6 Institute of Pathology, Faculty of Medicine and University Hospital Carl Gustav Carus, Technische Universität Dresden, Dresden, Germany; 7 Tumour- and Normal Tissue Bank, University Cancer Centre (UCC), University Hospital Carl Gustav Carus, Technische Universität Dresden, Dresden, Germany; 8 German Cancer Consortium (DKTK) Partner Site Berlin, Germany and German Cancer Research Center (DKFZ), Heidelberg, Germany; 9 Department of Radiooncology and Radiotherapy, Charité University Medicine Berlin, Berlin, Germany; 10 German Cancer Consortium (DKTK), Partner Site Essen, Germany and German Cancer Research Center (DKFZ), Heidelberg, Germany; 11 Department of Radiotherapy, University Hospital Essen, Medical Faculty, University of Duisburg-Essen, Essen, Germany; 12 German Cancer Consortium (DKTK), Partner Site Frankfurt, Germany and German Cancer Research Center (DKFZ), Heidelberg, Germany; 13 Department of Radiotherapy and Oncology, Goethe-University, Frankfurt, Germany; 14 German Cancer Consortium (DKTK), Partner Site Munich, Germany, German Cancer Research Center (DKFZ), Heidelberg, Germany; 15 Department of Radiation Oncology, Ludwig-Maximilians-Universität, Munich, Germany; 16 Clinical Cooperation Group, Personalized Radiotherapy in Head and Neck Cancer, Helmholtz Zentrum, Munich, Germany; 17 Department of Radiation Oncology, Medical University of Innsbruck, Innsbruck, Austria; 18 Department of Radiation Oncology, Technische Universität München, Munich, Germany; 19 Institute of Radiation Medicine (IRM), Helmholtz Zentrum München, Neuherberg, Germany; 20 German Cancer Consortium (DKTK), Partner Site Tübingen, Germany German Cancer Research Center (DKFZ), Heidelberg, Germany; 21 Department of Radiation Oncology, Faculty of Medicine and University Hospital Tübingen, Eberhard Karls Universität Tübingen, Tübingen, Germany; 22 German Cancer Research Center (DKFZ), Heidelberg, Germany

**Keywords:** Head and neck cancer, Predictive markers, Prognostic markers, Cancer imaging

## Abstract

Patients with locally advanced head and neck squamous cell carcinoma (HNSCC) may benefit from personalised treatment, requiring biomarkers that characterize the tumour and predict treatment response. We integrate pre-treatment CT radiomics and whole-transcriptome data from a multicentre retrospective cohort of 206 patients with locally advanced HNSCC treated with primary radiochemotherapy to classify tumour molecular subtypes based on radiomics, develop surrogate radiomics signatures for gene-based signatures related to different biological tumour characteristics and evaluate the potential of combining radiomics features with full-transcriptome data for the prediction of loco-regional control (LRC). Using end-to-end machine-learning, we developed and validated a model to classify tumours of the atypical subtype (AUC [95% confidence interval] 0.69 [0.53–0.83]) based on CT imaging, observed that CT-based radiomics models have limited value as surrogates for six selected gene signatures (AUC < 0.60), and showed that combining a radiomics signature with a transcriptomics signature consisting of two metagenes representing the hedgehog pathway and E2F transcriptional targets improves the prognostic value for LRC compared to both individual sources (validation C-index [95% confidence interval], combined: 0.63 [0.55–0.73] vs radiomics: 0.60 [0.50–0.71] and transcriptomics: 0.59 [0.49–0.69]). These results underline the potential of multi-omics analyses to generate reliable biomarkers for future application in personalized oncology.

## Introduction

Head and neck squamous cell carcinoma (HNSCC) is the 6th most common tumour entity worldwide^1^, arising within the mucous membranes of the mouth and throat with known risk factors being human papillomavirus (HPV) infection, smoking, and alcohol consumption^1^. Depending on the tumour site and staging, treatment may combine surgery, radiotherapy, and chemotherapy^2^. Locally advanced cases show a heterogeneous treatment response with 5-year overall survival (OS) of approximately 50%^1^. In the era of precision medicine, novel concepts for personalized treatment are developed, including combined treatment modalities or biomarker-guided radiotherapy dose prescription^3^. For this aim, novel biomarkers that reflect the heterogeneity of the tumours are required. An integration of independent omics layers, i.e. multi-omics, may hold particular value to identify accurate biomarkers^4^.

Four HNSCC molecular subtypes have been identified by gene expression studies^5^, providing insight into the etiological heterogeneity of the tumours^6^. The atypical, basal, classical, and mesenchymal subtypes have been respectively related to a strong HPV+ gene signature lacking EGFR amplification^6^, hypoxia and gene signatures of basal cells from the human airway epithelium^5,7^, tobacco intake^5^, and acquisition of stemness and migration^5,8^. In order to assess treatment response, different biological tumour characteristics have been considered and corresponding biomarkers were developed, for example based on cancer stem cell markers^9^, genes associated to proliferation and DNA repair^10^, radioresistance^11,12^, immune response^13^, hypoxia^14^, or epithelial–mesenchymal transition^15^. These biomarkers are commonly determined from biopsies or surgical specimens of the tumour. Radiological imaging is an additional source of information and may hold the potential to supplement molecular biomarkers for increased prognostic performance or to replace them, e.g., to avoid invasive biopsies or enable tumour assessment at additional time points.

Radiomics analyses perform a quantitative characterisation of medical images to derive image biomarkers, employing machine learning algorithms for diagnosis or the prognosis of treatment outcome^16^. The relationship between radiomics features and underlying biological mechanisms are not well understood^17^. Radiogenomics (imaging genomics) has emerged as a field whose objective is the association of genomics and molecular alterations within a tumour with quantitative features derived from a radiomics analysis and their integration. For HNSCC, CT texture features have been related to cell-cycle mutations^18^ and classification models for five sites of methylation as well as NSD1 mutation have been developed using CT-based radiomics^19^.

Here, we further explored the link between macroscopic radiomics features and the underlying tumour biology by analysing a well characterised multicentre cohort of patients with locally advanced HNSCC treated with primary radiochemotherapy (RCTx). Based on paired whole-transcriptome microarray and CT imaging data, we assessed (1) if radiomic features could represent the four molecular HNSCC subtypes, (2) if radiomics features could predict gene-based classifiers related to different biological tumour characteristics such as hypoxia, immune processes, epithelial–mesenchymal transition, and radiosensitivity, and (3) if the prognostic value of CT-based radiomics and whole-transcriptome data could be enhanced by their integration.

## Results

**Figure 1:**
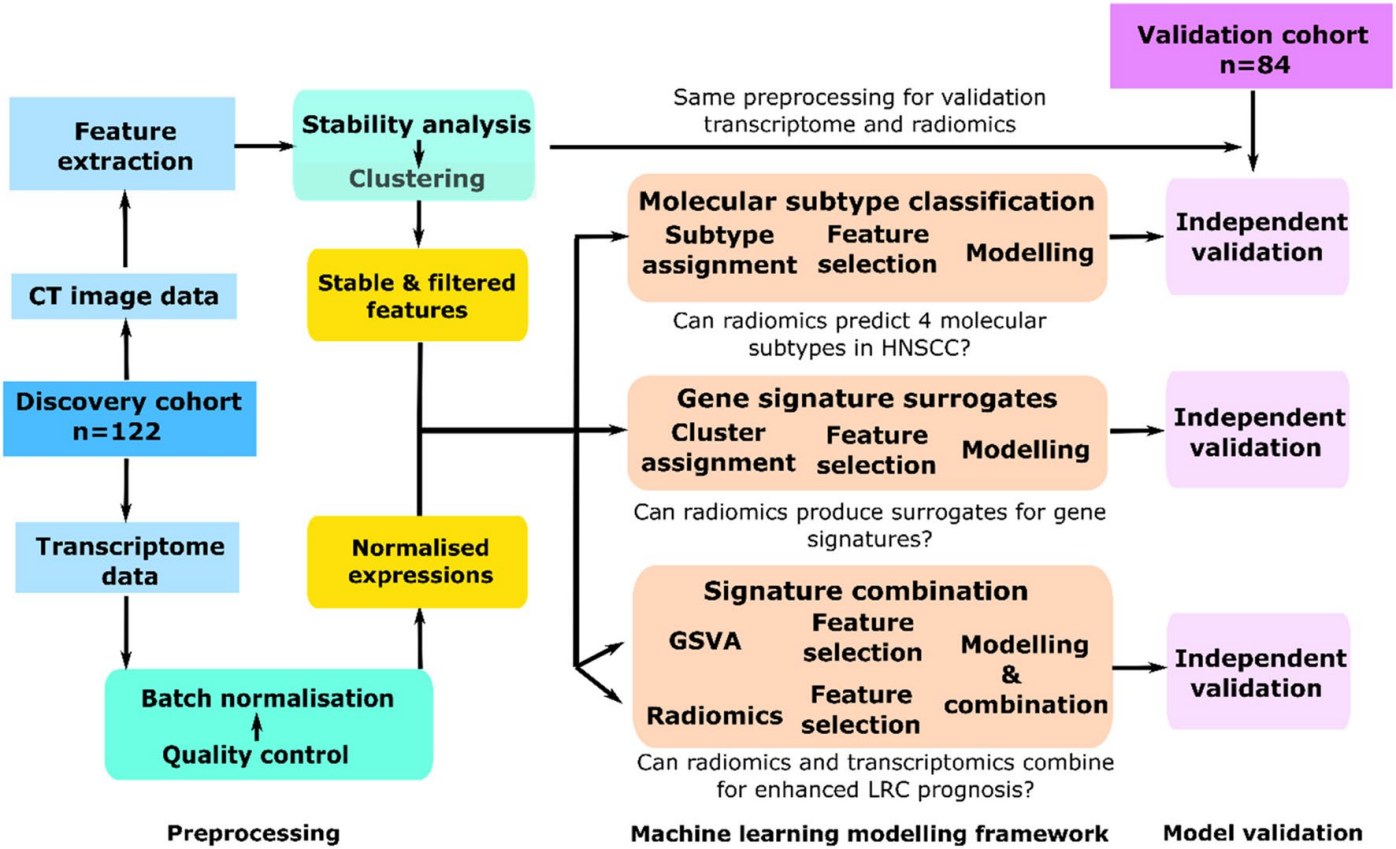
Overview of the study design. Pre-treatment CT and transcriptome data from patients with HNSCC are first pre-processed to obtain stable and filtered radiomics features and normalised gene expressions. For molecular subtype classification, molecular subtypes are assigned based on centroid data. Then radiomics features are selected through a machine learning pipeline to train OVA models for each subtype. For gene signature surrogates, binary cluster assignment is performed based on a k-means algorithm. Radiomics models are then trained to classify the gene signatures. For signature combination, a radiomics signature is developed and relevant metagenes are derived using GSVA. The identified features are combined to predict LRC. Subsequently, all models are independently validated.

Whole-transcriptome and pre-treatment CT data were obtained from a retrospective, multicentric cohort of 206 patients presenting with histopathologically confirmed, locally advanced HNSCC who were treated with primary RCTx. This cohort was split into a discovery cohort (n = 122) and a validation cohort (n = 84) to assess if radiomics can (1) identify the four molecular subtypes, (2) predict biological processes represented by different gene signatures, and (3) be integrated with full-transcriptome data for an improved prediction of LRC. Figure 1 presents the design of our study.

Clinical characteristics of both patient cohorts are presented in Table 1. Patients from the validation cohort presented with larger tumours, which were treated with marginally higher dose. Tumours in this cohort were more often found within the hypopharynx and larynx. Only the tumour volume was found to be significantly related to the endpoints of loco-regional control (LRC) and overall survival (OS) (Supplementary Tables 1, 2).

**Table 1:** Characteristics of clinical features for the discovery cohort (left) and validation cohort (right) along with p values for homogeneity tests between the cohorts.

Variables	Discovery cohort	Validation cohort	p value
	Median (range)	Median (range)	
GTV (cm^3^)	27.0 (4.4–175.8), missing: 0	40.5 (2.7–238.8) missing: 0	** < 0.001**
Age (years)	59.5 (39.2–80.2), missing: 0	56.0 (41.0–82.1) missing: 0	0.16
Total dose (Gy)	72.0 (69.2–74.0), missing: 0	72.0 (69.0–72.0) missing: 0	** < 0.001**
	**Number of 122 (%)**	**Number of 84 (%)**	
**Gender**			
0 (male)	99 (81.1)	68 (80.9)	1.0
1 (female)	23 (18.9)	16 (19.1)	
**Tumour site**			
Oropharynx	61 (50.0)	22 (26.2)	** < 0.001**
Hypopharynx	39 (32.0)	24 (28.6)	
Larynx	0 (0.0)	9 (10.7)	
Oral cavity	22 (18.0)	29 (34.5)	
**UICC stage (2010)**			
2	0 (0.0)	1 (1.2)	0.39
3	11 (9.1)	9 (10.7)	
4	111 (90.9)	72 (85.7)	
Missing	0 (0.0)	2(2.4)	
**cT stage**			
2	15 (12.3)	4 (4.8)	0.18
3	36 (29.5)	22 (26.2)	
4	71 (58.2)	58 (69.0)	
**cN stage**			
0	26 (21.3)	12 (14.3)	0.39
1	4 (3.3)	6 (7.1)	
2	86 (70.5)	61 (72.6)	
3	6 (4.9)	5 (6.0)	
**Grading**			
1	4 (3.3)	1 (1.2)	** < 0.001**
2	85 (69.7)	34 (40.5)	
3	30 (24.6)	30 (35.7)	
Missing	3 (2.4)	19 (22.6)	
**HPV16-DNA**			
0 (negative)	109 (88.5)	70 (83.3)	0.18
1 (positive)	12 (9.8)	8 (9.5)	
Missing	1 (0.7)	6 (7.2)	
**Alcohol**			
0 (no)	50 (41.0)	26 (31.0)	0.28
1 (regular)	70 (57.4)	35 (41.6)	
Missing	2 (1.6)	23 (27.4)	
**Smoking**			
0 (negative)	17 (13.9)	17 (20.2)	1.00
1 (positive)	105 (86.1)	67 (79.8)	
**Radiotherapy technique**			
3D-conformal	36 (29.5)	30 (33.7)	0.48
IMRT	84 (68.9)	54 (64.3)	
Missing	2 (1.6)	0 (0.0)	
**Dose schedule**			
Normofractionated	41 (33.6)	3 (3.6)	** < 0.001**
Hyperfractionated accelerated	66 (54.1)	81 (96.4)	
Missing	15 (12.3)	0 (0.0)	

*Abbreviations: GTV*: gross tumour volume, *UICC:* Union for International Cancer Control, *HPV16:* human papillomavirus type 16, *DNA:* deoxyribonucleic acid, *IMRT*: intensity-modulated radiotherapy.

### Radiomics can predict the atypical subtype

**Figure 2:**
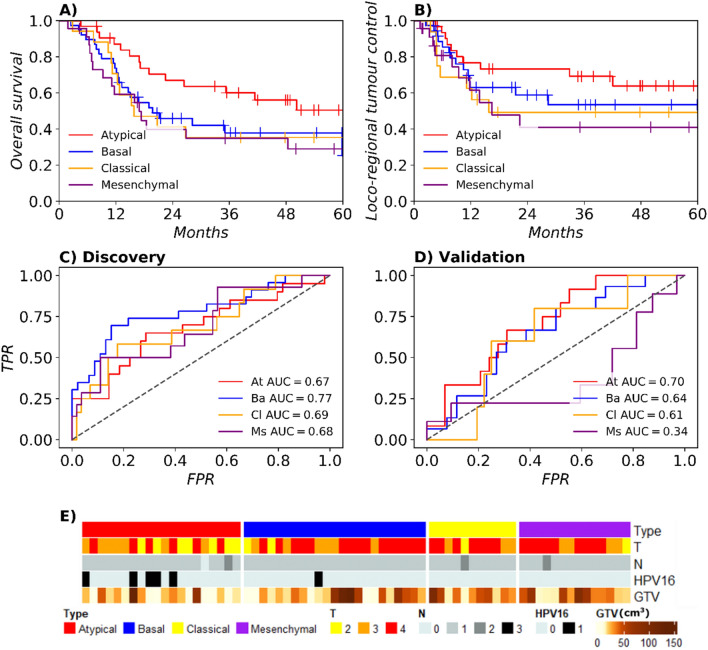
Outcome stratified by molecular subtypes and their classification using radiomics. Kaplan–Meier survival curves show that atypical tumours have the best prognosis for both OS (**A**) and LRC (**B**) with the other three subtypes having similar outcomes. The receiver-operating characteristics (ROC) curves with false positive rate (FPR) and true positive rate (TPR) show good AUC point-estimates in the discovery cohort (**C**). For the atypical subtype and to a lesser extent for the basal subtype, this performance is translated to the validation cohort (**D**). The associations of molecular subtypes with the clinical characteristics T stage, N stage, HPV16 status and gross tumour volume (GTV) are shown in (**E**) for the discovery cohort. Atypical tumours are smaller, have a lower T stage, and are more often HPV+.

Patients were classified into one of the four HNSCC molecular subtypes through correlation with published centroid data^5^: atypical (15.6%), basal (18.5%), classical (8.3%), and mesenchymal (11.2%). The remaining patients could not be classified due to either low correlation to all subtypes or similar correlations between two or more subtypes (46.4%). Association of molecular subtypes with OS and LRC is shown in Fig. 2A,B, respectively. Patients with tumours of the atypical subtype showed significantly higher OS compared to patients with other tumour subtypes (p = 0.039). These patients were more likely to have HPV+ tumours (p = 0.005) and lower T-stage (p = 0.040), as shown in Fig. 2E. Model performances using tumour site for all subtypes and the GTV and site for the atypical subtype were low, as shown in Supplementary Table 3. Clinical characteristics of patients per subtype are displayed in Supplementary Table 4.

Pre-treatment CT-derived radiomics features were computed, clustered, and used to train four one-versus-all (OVA) logistic regression models to distinguish between subtypes through an end-to-end modelling pipeline. Selected features and their scores across cross-validation folds of the discovery cohort are displayed in Supplementary Table 5 for each model. Receiver operating characteristic (ROC) curves for the models of each subtype are shown in Fig. 2C (discovery) and 2D (validation) alongside the point estimate area under the curve (AUC). As shown in Table 2, the atypical subtype had the highest performance with an AUC of 0.68 (95% confidence interval (CI) [0.52–0.79]) in the discovery cohort and was successfully validated with an AUC of 0.69 [0.53–0.83] in validation, accompanied by an f1-score of 0.47 [0.43–0.51] in discovery and of 0.53 [0.44–0.62] in validation. Cutoffs for the f1 scores are shown in Supplementary Table 6. This model was well-calibrated according to the Hosmer–Lemeshow (HL) test (discovery: p = 0.75, validation: p = 0.37) as shown in Supplementary Fig. 1A,B. Models for the other subtypes performed well in the discovery cohort but could not be validated.

**Table 2:** Model performance for molecular subtype classification: median area under the curve (AUC) and f1 score in discovery (disc) and validation (val) cohorts with 95% confidence intervals (CI) alongside the p value for calibration using the Hosmer–Lemeshow (HL) test in validation.

Positive class	AUC disc [95% CI]	AUC val [95% CI]	f1 disc [95% CI]	f1 val [95% CI]	HL val p value
Atypical	0.68 [0.52–0.79]	0.69 [0.53–0.83]	0.47 [0.43–0.51]	0.53 [0.44–0.62]	0.37
Basal	0.76 [0.62–0.88]	0.63 [0.44–0.80]	0.67 [0.52–0.80]	0.55 [0.40–0.68]	0.10
Classical	0.69 [0.51–0.84]	0.62 [0.28–0.81]	0.33 [0.31–0.36]	0.21 [0.11–0.28]	0.18
Mesenchymal	0.68 [0.51–0.84]	0.33 [0.12–0.58]	0.42 [0.35–0.48]	0.26 [0.11–0.38]	< 0.001
Atypical (HPV−)	0.70 [0.53–0.84]	0.74 [0.56–0.89]	0.42 [0.40–0.46]	0.54 [0.48–0.62]	0.15

*Abbreviations: HPV:* human papillomavirus.

**Figure 3:**
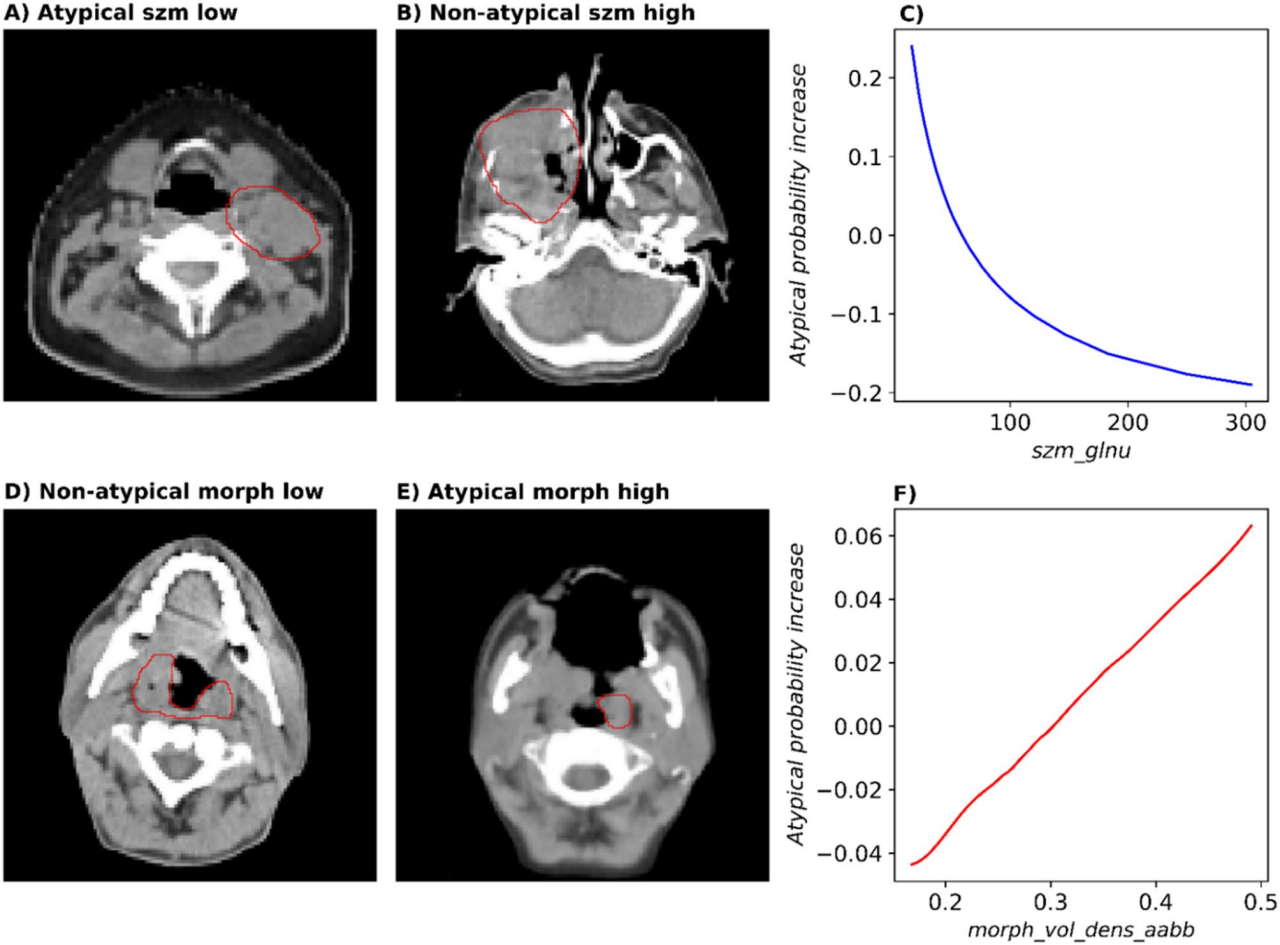
Representative pre-treatment CT slices (primary tumour in red) with high and low values of the two features from the radiomics model for the atypical subtype and accumulated local effects plot (ALE) in the discovery cohort. CT slices of patients with tumours that are characterised by low and high values of the texture feature grey level non-uniformity (siye zone matrix) are presented in panels (**A**,**B**), classified as atypical and non-atypical, respectively. A lower value of this feature represents a more spatially homogeneous tumour. The patient presented in panel (**D**) suffered from a non-atypical tumour with a low expression of the morphological feature volume density (axis-aligned bounding box). It showed a less regular shape than the atypical tumour of the patient in panel (**E**) with high feature expression. ALE plots (**C**,**F**) show difference in probability of being classified as atypical compared to an average patient for each feature value.

The selected radiomics features for the presented atypical model were the morphological feature morph_vol_dens_aabb (IBSI: PBX1) and texture feature szm_glnu (IBSI: JNSA), being, respectively, the fraction of a rectangular box around the tumour volume that is occupied by the tumour and the non-uniformity of the size of similar grey level zones. Table 3 shows transformation parameters and coefficients for the atypical model. A higher morph_vol_dens_aabb was related to higher odds of belonging to the atypical class, whereas a higher szm_glnu indicated lower odds. This implies that atypical tumours are more likely to be coarser and have larger spatial patterns, as they tend to be macroscopically more homogeneous and they tend to have a more regular shape, as visualized in Fig. 3.

**Table 3:** Information on features and intercept in the final logistic regression model for the atypical subtype: coefficients with 95% confidence intervals (CI) along with model p values, Yeo–Johnson (λ) and z-transform (z-shift and z-scale) parameters.

Feature	Coefficient [95% CI]	p value	λ	z-shift	z-scale
morph_vol_dens_aabb	0.114 [− 0.468 to 0.696]	0.69	− 0.5	0.246	0.046
szm_glnu	− 0.545 [− 1.166 to 0.076]	0.087	0	4.190	0.669
Intercept	− 0.968 [− 1.523 to − 0.413]	< 0.001	NA	NA	NA

Finally, to assess whether our model only predicts the HPV status, we assessed the observed features based on HPV16-DNA− tumours only. In a new OVA atypical model excluding HPV16-DNA+ tumours, the previously identified features retained their performance with an AUC of 0.70 [0.53–0.84] in discovery and 0.74 [0.56–0.89] in validation, as well as good calibration (Table 2, Supplementary Fig. 1C,D).

### CT radiomics shows limited value for predicting six gene classifiers related to different tumour characteristics of locally advanced HNSCC

Patients were classified into two classes for each of six different gene signatures: a 7-gene signature related to DNA repair and progression^10^, an 11-gene radiosensitivity signature^11^, a 12-gene immune signature^13^, a 15-gene hypoxia signature^14^, a 32-gene radiosensitivity signature^12^, and a 42-gene signature for epithelia-mesenchymal transition (EMT)^15^, with the aim to predict the classes using radiomics models. The selected features and their scores across cross-validation folds of the discovery cohort are displayed in Supplementary Table 7 for each gene signature.

None of the classifiers could successfully represent the gene signatures by a radiomics model in validation, as for all models, the lower end of the AUC’s 95% CI included the value 0.50 (Supplementary Table 8). Individual genes within the signatures showed low to moderate Spearman correlations with the radiomics features within the discovery cohort (Supplementary Fig. 2).

### Combining transcriptome data and CT-based radiomics improves LRC prediction

We assessed whether the integration of whole-transcriptome gene-expression data with CT-based radiomics features improves the prediction of LRC.

**Figure 4:**
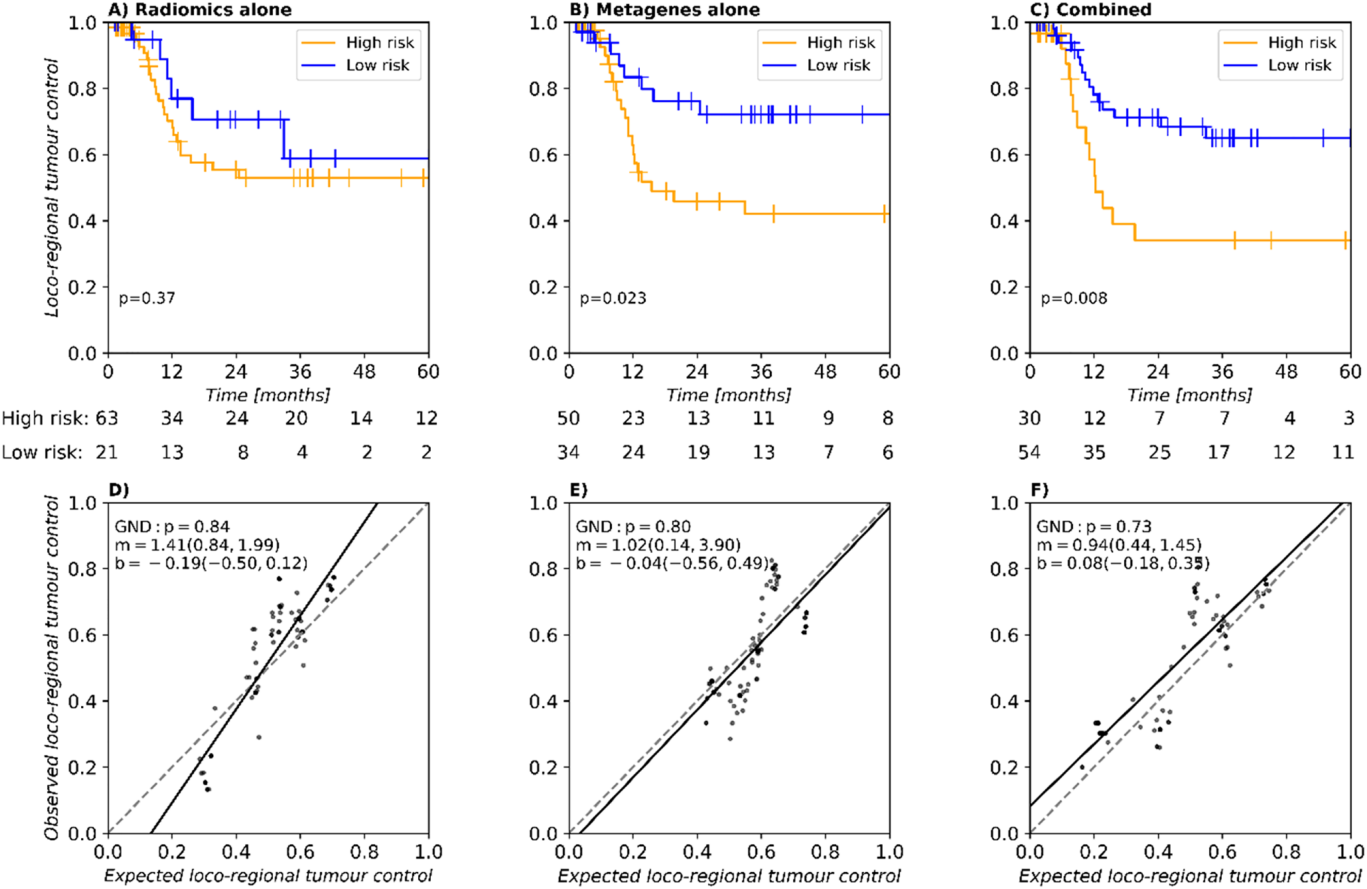
Stratification (**A**–**C**) and calibration (**D**–**F**) of the radiomics (left), metagene (centre), and combined model (left) for the prognostic value of loco-regional control (LRC) in the validation cohort. The radiomics model has a non-significant stratification. The metagene model achieves significant stratification and good calibration. When combining the radiomics signature and metagenes, a well-calibrated model with the best stratification in the validation cohort is obtained.

A radiomics signature was obtained based on a previously implemented workflow^20^. This signature consists of the tumour volume and the additional imaging feature log_stat_p90 (IBSI: 8DWT). Scores and hyperparameters for the selected features are shown in Supplementary Table 9. In Cox regression, the signature showed a median C-Index of 0.63 [0.54–0.72] on the discovery cohort and of 0.60 [0.50–0.71] on the validation cohort for LRC. Patient stratification was statistically significant (p < 0.001) in the discovery cohort but not in validation (p = 0.37) as shown in Supplementary Fig. 3A and Fig. 4A, respectively. Model calibration was successful in the discovery (Supplementary Fig. 3D) and validation cohorts (Fig. 4D).

To identify relevant characteristics from whole transcriptome data, Gene Set Variation Analysis (GSVA) was applied^21^. Metagenes representing the activation of gene sets related to hallmarks of cancer were derived from MsigDB^22^. On the development cohort, these metagenes were assessed for association with LRC in a feature-selection procedure. The gene set representing hedgehog signalling was selected alongside the gene set for E2F transcriptional targets. These gene sets consist of 35 and 200 genes presented in Supplementary Tables 10 and 11, respectively, some of which were related to a worse outcome in HNSCC^23^, chemoresistance^23^, radioresistance^24^, and proliferation^25^ for the hedgehog metagene. The E2F transcriptional targets metagene contains genes associated with progression in advanced stages^26^, worse outcome^27^, or chemoresistance^28^. In prognostic modelling for LRC, the two metagenes achieved a median C-Index of 0.59 [0.51–0.67] on the discovery cohort and 0.59 [0.49–0.69] in validation. Patient stratification was significant in the discovery cohort (p = 0.022) and in validation (p = 0.023) as shown in Supplementary Fig. 3B and Fig. 4B, respectively. Calibration was successful in the discovery cohort (Supplementary Fig. 3E) and in validation (Fig. 4E).

Finally, the two metagenes were combined with the radiomics signature to train a Cox model on the entire discovery cohort. Compared to the two individual models presented before, this integrated model yielded a moderately improved median C-Index of 0.65 [0.56–0.74] for the discovery cohort and of 0.63 [0.55–0.73] for validation. Moreover, using a risk threshold of 1.502, a significant difference in LRC between the two risk groups was visible in both discovery (p < 0.001) and validation (p = 0.008), as can be observed from Supplementary Fig. 3C and Fig. 4C, respectively. Patients assigned to the high-risk group tended to have larger values of the radiomics feature log_stat_p90, larger tumour volume, and highly expressed hedgehog metagene, while having lower values of the E2F metagene as compared to the low-risk group (Supplementary Fig. 4). Calibration was successful in discovery (Supplementary Fig. 3F) and validation (Fig. 4F). Model details are presented in Table 4. Justification of the proportional hazards (PH) assumption for the three models is shown in Supplementary Table 12.

**Table 4:** Information on features of the multivariable Cox regression model integrating CT radiomics features and the two selected metagenes: hazard ratio (HR) (95% CI) along with model p values, Yeo–Johnson parameter (λ) and transformation parameters for z-transform (z-shift and z-scale).

Feature	HR [95% CI]	p value	λ	z-shift	z-scale
GTV (cm^3^)	1.196 [0.897–1.594]	0.22	0	10.170	0.824
Log_stat_p90	1.393 [1.036–1.875]	0.028	0	1.256	0.499
E2F_targets	0.774 [0.580–1.033]	0.081	1	− 0.014	0.416
Hedgehog_signalling	1.329 [0.990–1.785]	0.058	0	− 0.055	0.280

To ascertain the role of the log_stat_p90 feature, we trained another model using the GTV and the metagenes. The performance on the discovery cohort was 0.63 [0.55–0.72] and 0.62 [0.53–0.72] in validation. Stratification was significant in discovery (p < 0.001), but only showed a trend in validation (p = 0.074) compared to the model with log_stat_p90. Calibration was successful as well in both discovery (p = 0.73) and validation (p = 0.98). Kaplan Meier curves and calibration plots for the model are shown in Supplementary Fig. 5.

## Discussion

Within the present study, we investigated the relationship between macroscopic radiomics features and gene-based information on biological tumour characteristics within locally advanced HNSCC. Using CT-derived radiomics, whole-transcriptome data, and machine learning techniques, we (1) developed and validated a model to differentiate the atypical subtype from the other subtypes, (2) observed that radiomics models had limited value as surrogates for six selected gene signatures, and (3) demonstrated the added value of combining full-transcriptome data with a radiomics signature for the prediction of LRC. Overall, this study comprises one of the most extensive multi-omics analyses combining imaging and transcriptome data for patients with locally advanced HNSCC so far.

Several studies have combined radiomics signatures with other types of data: the combination with clinical data^29^ and of different imaging modalities, e.g. CT and PET^30^, has been shown to improve prognostic value. Radiogenomics within HNSCC so far focused mostly on developing and validating signatures for HPV status prediction: Bogowicz et al. derived a CT radiomics signature with a validation AUC of 0.80 on patients with definitive radiochemotherapy^31^, showing that HPV+ tumours appear more homogeneous on CT. Some HNSCC studies dealt with the association of radiomics texture features to somatic mutations, e.g. Zwirner et al. correlated somatic mutations of FAT1 to decreased intra-tumour heterogeneity^18^. Huang et al.^19^ predicted the four molecular subtypes using patients with pre-treatment CT from The Cancer Genome Atlas (TCGA), with the atypical subtype displaying an AUC of 0.65, but was not restricted to locally advanced HNSCC patients and the subtype models were not validated.

Atypical HNSCC was shown to be associated to HPV positivity^5,32^. In our study, the proportion of HPV16-DNA positive tumours within the atypical group was significantly higher than for the other subtypes. These tumours were visually more homogeneous than the other subtypes as reflected by the selected radiomics features. The selected morphological and texture features volume density (axis-aligned bounding box) and grey level non-uniformity (size zone matrix) represent the volume fraction that the tumour volume has within a corresponding bounding box and the non-uniformity of the grey levels in the size zone matrix, respectively. A higher volume density (axis-aligned bounding box) would represent a more spatially regular, box-like volume of the tumour. A higher grey level non-uniformity (size zone matrix) would indicate a more inhomogeneous tumour in grey levels with bigger spatial patterns as more zones have grey levels that predominate over others. To confirm that our radiomics model does not only predict the HPV status, it was retrained on the HPV- subpopulation, achieving a similarly good performance. Atypical tumours are described as lacking epithelial growth factor receptor (EGFR) amplification^5,6,19^, a prominent oncogene in HNSCC, therefore possibly making EGFR-targeted therapies such as Cetuximab less effective. They are also described as being more radiosensitive than the other subtypes, even when they are HPV−^33^. A model predicting the atypical subtype, especially for HPV− tumours, may thus be clinically relevant. Radiomics models to identify the other three subtypes (basal, classical, and mesenchymal) could not be successfully validated. This might indicate that the three remaining subtypes are more similar from a CT radiomics point of view compared to the atypical subtype.

Within this study, non-contrast-enhanced CT-based radiomics showed limited capabilities to predict the classification of the 6 selected gene signatures related to different molecular tumour processes within HNSCC, as our radiomics models could not be validated. This might be due to the gene signatures reflecting microscopic processes that are not readily observable at the macroscopic image level where CT-based radiomics operates. While studies have been conducted to predict hypoxia using radiomics within HNSCC, they employed functional imaging e.g. PET, either totally^34^ or partially^35^. These studies showed low to moderate correlations of radiomics features with gene-based hypoxia markers^36^. MRI has been used to study hypoxia in glioblastoma^37^ and prostate cancer^38^, suggesting that other imaging modalities might be more appropriate for this task.

As the CT-based radiomics features showed low correlations to the considered gene signatures, the combination of such non-redundant data may be useful to improve biomarkers for the prognosis of treatment outcome. Here, we observed an improvement in discrimination through the combination of a developed CT-based radiomics signature with two GSVA-derived^21^ metagenes representing the activation of the hedgehog pathway and E2F transcriptional targets genes from MsigDB^22^, achieving significant stratification and good model calibration. Higher values of the hedgehog metagene were related to a higher risk of a loco-regional recurrence, which is in agreement with the literature, where higher expression of this pathway in HNSCC has been related to worse prognosis^25^. Its inhibition through antagonists such as cyclopamine or vismodegib has been shown to suppress proliferation^39^, stromal repopulation after radiotherapy through GLI1 expression^24^, and to aid in sensitization to radiotherapy through GLI1 and IAP^40^. Transcription factors encoded by the E2F transcriptional target genes are related to DNA repair and cell-cycle regulation. E2F transcriptional targets expression has been used to partition HNSCC into subtypes^41^ with HPV+ tumours tending to show high values of E2F-regulated genes, while HPV− tumours tended to show lower values, which might relate to the observation that higher values of the E2F transcriptional targets metagene were associated with a lower risk of LRC in our model.

This retrospective study has limitations. Transcriptome data originate from single biopsies of the tumours, which likely do not reflect the heterogeneous transcriptome within locally advanced tumours^42^. Repeated biopsies at different locations within the tumour, however, were not available. Only pre-treatment CT imaging data were available for radiomics analysis, while other imaging modalities, for example PET or MRI, may show stronger associations to the underlying tumour biology. For the molecular subtype classification task, the limited amount of discovery subjects to train the models made us consider an OVA approach instead of a potentially more clinically-useful multiclass classifier. Furthermore, the limited number of classical-type tumours within the validation cohort prevented the assessment of the OVA model performance due to wide confidence intervals. The observed differences in clinical parameters between the discovery and validation cohort may impact the validation of the developed models and while image and batch normalisation were performed, metadata missingness may mask confounding effects for the radiomics models like the tube voltage, reconstruction kernels or exposure time.

In conclusion, we have evaluated three different aspects of the relationship between macroscopic CT-based radiomics and whole-transcriptome data in patients with HNSCC: (1) The atypical molecular subtype could be well classified by a radiomics approach, (2) representing gene signatures by radiomics features was not successful, and (3) a combination of a radiomics signature with two transcriptome metagenes led to an improved prediction of LRC. These results underline the potential of multi-omics analyses to generate reliable biomarkers for application in personalized oncology. In the future, we aim to validate our findings within the prospective HNPrädBio study of the DKTK-ROG (www.clinicaltrials.gov, NCT02059668), before potential application in an interventional clinical trial for personalised treatment of locally advanced HNSCC.

## Materials and methods

### Patient cohort

The present multicentric retrospective study was conducted on 206 patients for whom treatment-planning CT imaging and whole-transcriptome data of a tumour biopsy were available. All patients were diagnosed with histopathologically confirmed locally advanced HNSCC and underwent primary RCTx with curative intent. Radiation dose (median 72 Gy, range [69–74 Gy]) was prescribed to the tumour region consisting of the tumour primary and grossly involved lymph nodes. Concomitant cisplatin (95.5%) or mitomycin C (4.5%) were applied in combination with 5-fluorouracil. Included patients were part of several previously published retrospective studies^9,43–45^. Patients were allocated to a discovery (n = 122) and a validation cohort (n = 84) according to these studies. Patients of the discovery cohort were treated at one of six partner sites of the German Cancer Consortium—Radiation Oncology Group (DKTK-ROG) between 2006 and 2010^9^. In the validation cohort, 39 patients were treated at the University Hospital Dresden (UKD) between 2002 and 2014^43^ and 44 patients were treated within a prospective imaging trial (NCT00180180) at the UKD between 2006 and 2013^44,45^. Inclusion and exclusion criteria were presented previously^9^. Ethical approval for multicentre retrospective analyses of clinical, imaging and biological data was obtained from the Ethics Committees of all DKTK partner sites. The endpoints LRC and OS were considered and calculated from the first day of RCTx until the day of the corresponding event or censoring.

Formalin-fixed paraffin-embedded (FFPE) blocks of the primary tumour biopsies were collected centrally at the DKTK partner site Dresden for extraction of total RNA for whole-transcriptome analysis as described previously^46^. HPV16-DNA status was obtained as described previously^9^. CTs and contours of the gross tumour volume (GTV) were collected for radiomics analyses. The GTV was defined and segmented within the CT as the visible tumour primary and grossly involved nodes as specified in previous studies^9^.

Ethical approval for the multicentre retrospective analyses of clinical, imaging and biological data was obtained from the Ethics Committee at the Technische Universität Dresden, Germany (EK397102014) and from the Ethics Committees of all DKTK partner sites. The requirement for individual informed consent was waived owing to the retrospective nature of the study. All methods were performed in accordance with the relevant guidelines and regulations.

### Microarray data analysis

Whole-transcriptome analysis was conducted as described in a previous study^46^, using the Affymetrics Human Transcriptome Array (HTA) 2.0 (Thermo Fisher Scientific Inc., Waltham, MA, USA). Quality control procedures were performed on the microarray probe-level intensity files using Transcriptome Analysis Console (TAC) (Applied Biosystems, Waltham, Massachusetts, USA). Signal data were normalised using the Signal Space Transformation alongside with the Robust Multiarray Average method (SST-RMA)^47^. ComBat normalisation^48^ was subsequently performed to correct for systematic variability within our data arising from different experimental conditions between the different contributing studies^9,43–45^. Signal intensities were then filtered for coding genes that had a gene annotation, resulting in 25328 gene expression features.

### Image data pre-processing, feature extraction and stability analysis

Patients received a CT scan for treatment planning prior to radiotherapy. Acquisition and reconstruction parameters are summarized in Supplementary Table 13. The GTV was delineated in each scan by experienced radiation oncologists. Voxels in each CT volume were resampled to an isotropic size of 1.0 × 1.0 × 1.0 mm^3^ using cubic splines to compensate for differing voxel spacing and slice thickness between centres. Intensity values for the CT volumes were restricted to the range between −150 and 180 Hounsfield Units (HU) as the GTVs included air cavities and bone regions that had to be excluded. A set of Laplacian of Gaussian (LoG) filters with 5 different kernel widths (1, 2, 3, 4 and 5 mm) were applied individually to the base image and averaged to a single image to quantify characteristics such as edges (sharp transitions in image intensities) or blobs (gross image details).

From the base image and the LoG-transformed image, a set of 18 statistical, 2 local-intensity based, 29 morphological, 37 intensity-histogram-based, and 137 texture-based features were extracted from the GTV leading to 446 features per patient. The entire image pre-processing pipeline was implemented according to the Image Biomarker Standardisation Initiative (IBSI)^49^ using the publicly-available MIRP Python package^50^. Feature computation parameters are reported in Supplementary Table 14.

Image-augmentation strategies were employed to filter non-robust features^51^. The images were rotated (−4°, −2°, 0°, 2°, 4°) and the size of the GTV segmentation altered (−20%, −10%, 10%, 20%) in the discovery cohort, producing 20 images per patient from which to analyse feature stability through the intraclass correlation coefficient (ICC). Features for which the lower bound of the 95% confidence interval (CI) of the ICC fell below 0.75^52^ were considered unstable and subsequently dropped for both cohorts. Morphological and texture features from the LoG response map were dropped.

To correct for possible bias in features affected by differences in scanner types, convolution kernels and other parameters that might differ between centres, we performed a non-parametric ComBat correction with a reference batch^53^. PCA analysis of the statistical, intensity histogram-based, and texture features was performed and consensus clustering of the PCA features conducted to identify a reference cluster of patients and outlier patients using ConsensusClusterPlus^54^, with parameters shown in Supplementary Table 15. The reference cluster was defined as the biggest number of patients that did not change clusters across augmenting cluster number k (Supplementary Figs. 6, 7). ComBat adjustment was conducted on the outlier patients with the reference patients as a reference batch.

### Molecular subtype classification with CT-based radiomics

In order to classify the tumours into one of the four molecular subtypes, 838 gene expression features were selected according to previous studies^5^. Gene expression profiles were median-centered for each patient and the Pearson correlation to the published centroid data of each subtype was calculated and interpreted as reported previously^55^. Patients whose largest correlation coefficient was smaller than 0.2 or whose difference between the two highest coefficients was 0.2 or less were dropped, not being classified as any subtype or having an ambiguous classification between two subtypes, respectively. After assigning the subtypes, the Kaplan–Meier estimator was used to assess their association with the endpoints LRC and OS.

To develop radiomics-based subtype-classification models, radiomics features were clustered by hierarchical clustering with complete linkage within the discovery cohort to reduce redundancy. The distance metric was defined as 1 − |ρ| (Spearman correlation coefficient ρ) with the distance metric threshold to form the clusters being 0.3. Cluster representatives were features that had the highest within-cluster average Spearman correlation. Radiomics representatives in the discovery cohort were then transformed through a Yeo–Johnson transform and standardised through a z-transform and transformation parameters were transferred to the validation cohort. Afterwards, feature selection was performed based on minimum redundancy maximum relevance (MRMR) within 33 repetitions of threefold cross-validation of the discovery cohort. Features were aggregated across cross-validation folds and ranked based on the enhanced Borda score. Features across CV folds were determined based on Bayesian hyperparameter optimisation applying a sequential model-based optimisation algorithm^56^ on a logistic regression model on each fold. The final model was then trained on the whole discovery cohort using the highest-ranking features across CV folds and with the median number of features across CV folds. Models were subsequently validated on the validation cohort. This process was repeated four times, each with a different subtype as the positive class and the other three aggregated together as the negative class. This end-to-end pipeline was implemented using the Familiar package^57^.

### CT-based radiomics surrogates of biological characteristics

A systematic search across PubMed and Google Scholar was conducted to identify validated gene signatures in patients with locally advanced HNSCC treated with primary radiochemotherapy to subsequently explore the capabilities of CT-based radiomics to produce surrogates of such signatures.

Out of the 21 found studies and signatures, one gene signature was selected for each of the following mechanisms: DNA-repair, radioresistance, immune processes, hypoxia and epithelial–mesenchymal transition. In case more than one signature was found per mechanism, the signature defined on the cohort with the highest number of patients was included^58^. Chosen signatures were a 7-gene signature associated with proliferation and DNA-repair^10^, an 11-gene signature for radioresistance^11^, a 12-gene signature for immune processes^13^, a 15-gene hypoxia-associated signature^14^, and a 42-gene signature for epithelial–mesenchymal transition^15^. A well-known 31-gene radiosensitivity signature identified in a meta-analysis NCI-60 cell lines was also included^12^.

To derive radiomics surrogates, binary classes were created within the discovery cohort using k-means clustering on the gene expressions of each signature. Class assignment within the validation cohort was conducted by computing the Euclidean distance between the data points in the validation cohort and each class centroid from the discovery cohort, assigning the class represented by the closest centroid. Surrogate radiomics signatures were then created similarly as for molecular subtype classification.

### Derivation of signatures prognostic for LRC and their combination

In order to derive a CT-based radiomics signature prognostic for LRC, we first included the gross tumour volume as it is a known prognostic clinical variable^59^ and was also significantly associated with LRC in our discovery cohort (Supplementary Table 1). Afterwards, features that had an absolute Spearman ρ above 0.5 with the volume were discarded. Clustering of features and all subsequent steps were then conducted similarly as for molecular subtype classification. Model building was conducted using the Cox model, with the GTV always included as a model feature across CV folds.

To identify relevant characteristics from whole transcriptome data, Gene Set Variation Analysis (GSVA) was employed through the GSVA R package^21^. GSVA transforms data of selected gene sets into patient-specific pathway-level metagenes. We used 50 initial gene sets of hallmarks of cancer from MsigDB^22^. GSVA metagenes were computed separately for the discovery and validation cohorts to avoid information leakage and using the signed maximum deviation from 0 (Supplementary Table 16) as a metric, as proposed for gene sets from MsigDB^21^.

GSVA metagenes were filtered based on a Google Scholar search to discard pathways not known to be related to HNSCC (Supplementary Table 17). Subsequent transformation, normalisation, feature selection and model building steps were conducted similarly to the molecular subtype classification. Model building was conducted using the Cox model.

Integration of the radiomics signature and identified metagenes was performed by training a multivariable Cox model on the entire discovery cohort, including the identified radiomics features and metagenes, and validating it on the validation cohort.

### Statistical analyses

LRC and OS time-to-event endpoints were calculated from the first day of RCTx to the day of event or censoring. They were compared between patient subgroups using the log-rank test. Clinical categorical variables and subtype proportions were compared between discovery and validation cohorts through the ${\chi }^{2}$ test or the exact Fisher test for categorical variables with less than 10 cases. Continuous variables were compared using the Mann–Whitney-*U* test. All tests were conducted two-sided at p = 0.05 level of significance on R software version 4.0.5 (R Core Team, 2021). For univariate association of clinical features with the LRC and OS endpoints, missing values were imputed by their median value for numerical variables and by their mode for categorical variables except for alcohol consumption and smoking status. These two features were transformed into two binary features, respectively representing positivity and non-availability. The following categorical variables were binarized: cT stage (0 for cT < 4 and 1 for cT = 4), cN stage (0 for cN < 2 and 1 cN ≥ 2), Grading (0 for Grading ≤ 2 and 1 for Grading > 2) and UICC stage (0 for UICC < 4, 1 for UICC = 4).

Subtype classification of radiomics models was assessed through the AUC and the f1 score due to imbalance within the classes. Reference values (Supplementary Section: reference values for f1 scores), indicating the expected value for a random model that maximises the f1 score, are provided per subtype and cohort (Supplementary Table 18). Cutoffs for the f1 score were decided based on the cutoff with median f1 score above the reference value and with highest lower end of the 95% bootstrap CI on 600 bootstraps of the discovery cohort. AUC and accuracy were used to assess the performance for the gene signature classification, with accuracy threshold at 0.50. Median and 95% bootstrap CI are reported. Model calibration was computed through the Hosmer–Lemeshow test (HL test).

Proportional hazards assumption of the Cox models was assessed through the ${\chi }^{2}$ test of the Schoenfeld residuals. The prognostic value of Cox proportional hazard models for LRC was assessed through the C-Index, with median value alongside its 95% bootstrap CI reported. Patients were stratified into high and low risk groups using the optimised threshold of the predicted risk score on the discovery cohort obtained with the familiar package^57^. Model calibration was conducted at 24 months and evaluated based on the Greenwood-Nam-d’Agostino test (GND test)^60^, and by assessing fit and slope of the linear fit.

## Supplementary Information

Supplementary Information 1.

Supplementary Information 2.

## Data Availability

All data needed to evaluate the conclusions of the paper are present in the paper and/or the Supplementary Materials and auxiliary files. Model files are updated to https://github.com/oncoray/Radiogenomics. The familiar package used for the machine-learning workflow is available in CRAN.
