# Supplementary material for: Integrated radiogenomics analyses allow for subtype classification and improved outcome prognosis of patients with locally advanced HNSCC
Source: Sci Rep. 2022 Oct 6;12:16755. doi: 10.1038/s41598-022-21159-7 (PMC9537286; doi:10.1038/s41598-022-21159-7)
Supplement: Supplementary file 2 — Supplementary Information 2. [file 41598_2022_21159_MOESM2_ESM.docx]

**Supplementary Materials: Integrated radiogenomics analyses allow for subtype classification and improved outcome prognosis of patients with locally advanced HNSCC**

Asier Rabasco Meneghetti, Alex Zwanenburg, Annett Linge, Fabian Lohaus, Marianne Grosser, Gustavo B. Baretton, Goda Kalinauskaite, Inge Tinhofer, Nika Guberina, Maja Guberina, Panagiotis Balermpas, Jens von der Grün, Ute Ganswindt, Claus Belka, Jan C. Peeken, Stephanie E. Combs, Simon Böke, Daniel Zips, Esther G.C. Troost, Mechthild Krause, Michael Baumann and Steffen Löck*

**Supplementary Table 1:** Clinical feature associations with loco-regional control (LRC) in the discovery (disc) and validation (val) cohorts measured through the median concordance index (C-index) and its 95% confidence interval (CI).

| **Feature** | **C-index disc [95% CI]** | **C-index val [95% CI]** |
| --- | --- | --- |
| GTV | 0.59 [0.51-0.67] | 0.61 [0.51-0.72] |
| Age | 0.54 [0.46-0.62] | 0.55 [0.47-0.61] |
| Total Dose | 0.54 [0.45-0.61] | 0.54 [0.48-0.62] |
| Gender | 0.53 [0.48-0.57] | 0.43 [0.38-0.50] |
| Tumour site | 0.56 [0.47-0.62] | 0.53 [0.44-0.63] |
| UICC Stage (2010) | 0.54 [0.48-0.58] | 0.54 [0.46-0.60] |
| cT | 0.56 [0.47-0.62] | 0.54 [0.46-0.61] |
| cN | 0.54 [0.48-0.60] | 0.59 [0.50-0.67] |
| Grading | 0.52 [0.48-0.55] | 0.49 [0.47-0.55] |
| p16 | 0.54 [0.48-0.59] | 0.52 [0.46-0.56] |
| HPV16 DNA | 0.54 [0.48-0.58] | 0.51 [0.46-0.56] |
| Alcohol positive | 0.53 [0.47-0.58] | 0.52 [0.41-0.61] |
| Smoking positive | 0.53 [0.48-0.56] | 0.58 [0.51-0.63] |

**Supplementary Table 2:** Clinical feature associations with overall survival (OS) in the discovery (disc) and validation (val) cohorts measured through the median concordance index (C-index) and its 95% confidence interval (CI).

| **Feature** | **C-index disc [95% CI]** | **C-index val [95% CI]** |
| --- | --- | --- |
| GTV | 0.62 [0.55-0.68] | 0.62 [0.53-0.70] |
| Age | 0.52 [0.45-0.60] | 0.55 [0.47-0.61] |
| Total Dose | 0.52 [0.46-0.59] | 0.53 [0.48-0.59] |
| Gender | 0.52 [0.47-0.58] | 0.49 [0.43-0.56] |
| Tumour site | 0.54 [0.47-0.62] | 0.55 [0.47-0.62] |
| UICC Stage (2010) | 0.53 [0.48-0.59] | 0.54 [0.49-0.57] |
| cT | 0.56 [0.49-0.63] | 0.55 [0.48-0.61] |
| cN | 0.54 [0.48-0.58] | 0.61 [0.52-0.64] |
| Grading | 0.49 [0.47-0.52] | 0.49 [0.47-0.54] |
| p16 | 0.55 [0.48-0.58] | 0.50 [0.45-0.54] |
| HPV16 DNA | 0.52 [0.49-0.55] | 0.49 [0.45-0.53] |
| Alcohol positive | 0.51 [0.45-0.58] | 0.52 [0.45-0.59] |
| Smoking positive | 0.50 [0.45-0.54] | 0.53 [0.47-0.59] |

**Supplementary Table 3:** Performances of the models for subtype prediction using the tumor site for all tumors and the tumor volume (GTV) and site for the atypical subtype within the discovery (disc) and validation (val) cohorts. Results are presented with median AUC and 95% confidence interval (CI).

| **Subtype** | **Feature** | **AUC disc [95% CI]** | **AUC val [95% CI]** |
| --- | --- | --- | --- |
| **Atypical** | Site | 0.57 [0.42-0.69] | 0.52 [0.34-0.75] |
| **Atypical** | Site + GTV | 0.65 [0.51-0.79] | 0.57 [0.39-0.75] |
| **Basal** | Site | 0.62 [0.48-0.73] | 0.59 [0.42-0.74] |
| **Classical** | Site | 0.60 [0.47-0.74] | 0.60 [0.40-0.83] |
| **Mesenchymal** | Site | 0.53 [0.35-0.69] | 0.46 [0.28-0.66] |

**Supplementary Table 4:** Clinical factors by subtype and p-values for homogeneity tests. Homogeneity of continuous variables is reported via the Kruskal-Wallis test.

|  | **Atypical (n=32)** | **Basal (n=38)** | **Classical (n=17)** | **Mesenchymal (n=23)** |  |
| --- | --- | --- | --- | --- | --- |
| **Variable** | **Median (range)** | **Median (range)** | **Median (range)** | **Median (range)** | **p-value** |
| **GTV (cm^3^)** | 21.6 (4.55-129.0) | 41.4 (1.27-164.0) | 30.1 (7.29-174.0) | 37.5 (10.8-163.0) | 0.12 |
| **Age (years)** | 59.6 (46.0-80.9) | 54.0 (39.2-82.1) | 63.0 (45.0-76.2) | 55.0 (45.0-77.8) | 0.13 |
| **Dose (Gy)** | 72.0 (70.0-72.0) | 72.0 (70.0-72.0) | 72.0 (70.0-74.4) | 72.0 (70.0-72.0) | 0.29 |
|  | **Number of 32 (%)** | **Number of 38 (%)** | **Number of 17 (%)** | **Number of 23 (%)** |  |
| **Gender** |  |  |  |  | 0.60 |
| Male  Female | 27 (84.3)  5 (15.7) | 28 (73.7)  10 (26.3) | 14 (82.4)  3 (17.6) | 20 (87.0)  3 (13.0) |  |
| **Tumour Site** |  |  |  |  | 0.49 |
| Oral  Hypopharynx  Larynx  Oropharynx | 7 (21.8)  10 (31.3)  2 (6.25)  13 (40.6) | 12 (31.6)  7 (18.4)  1 (2.6)  18 (47.4) | 1 (5.9)  6 (35.3)  0 (0.0)  10 (58.8) | 5 (21.7)  7 (30.4)  0 (0.0)  11 (47.9) |  |
| **cT** |  |  |  |  | 0.30 |
| 2  3  4 | 8 (25.0)  10 (31.3)  14 (43.7) | 4 (10.5)  10 (26.3)  24 (63.2) | 1 (5.9)  6 (35.3)  10 (58.8) | 1 (4.3)  6 (26.1)  16 (69.6) |  |
| **cN** |  |  |  |  | 0.42 |
| 0  1  2  3 | 4 (12.5)  2 (6.25)  24 (75.0)  2 (6.25) | 11 (28.9)  2 (5.3)  24 (63.1)  1 (2.7) | 1 (5.9)  0 (0.0)  14 (82.3)  2 (11.8) | 4 (17.4)  0 (0.0)  17 (73.9)  2 (8.7) |  |
| **Grading** |  |  |  |  | 0.85 |
| 1  2  3  Missing | 0 (0.0)  19 (59.4)  8 (25.0)  5 (15.6) | 2 (5.3)  24 (63.1)  8 (21.1)  4 (10.5) | 0 (0.0)  11 (64.7)  5 (29.4)  1 (5.9) | 0 (0.0)  13 (69.6)  7 (30.4)  0 |  |
| **HPV16 DNA** |  |  |  |  | **0.04** |
| 0 (Negative)  1 (Positive)  Missing | 24 (75.0)  7 (21.9)  1 (3.1) | 36 (94.6)  1 (2.7)  1 (2.7) | 14 (82.3)  1 (5.9)  2 (11.8) | 21 (91.4)  1 (4.3)  1 (4.3) |  |
| **Alcohol** |  |  |  |  | 0.55 |
| 0 (Negative)  1 (Regular)  Missing | 13 (40.6)  16 (50.0)  3 (9.4) | 19 (50.0)  17 (44.6)  2 (5.4) | 5 (29.4)  10 (58.8)  2 (11.8) | 7 (30.4)  12 (92.2)  4 (17.4) |  |
| **Smoking** |  |  |  |  | 0.71 |
| 0 (Negative)  1 (Positive) | 4 (12.5)  28 (87.5) | 6 (15.8)  32 (84.2) | 1 (5.9)  16 (94.1) | 4 (17.4)  19 (82.6) |  |

*Abbreviations: GTV: gross tumour volume, UICC: Union for International Cancer Control, HPV16: Human papillomavirus type 16, DNA: deoxyribonucleic acid*

**Supplementary Table 5:** Selected features with enhanced Borda scores and occurrences across CV folds of the discovery cohort for each subtype classification model. All features were selected using the maximum-relevance minimum-redundancy (MRMR) selection algorithm. The only hyperparameter was the number of selected features, which was optimized through Bayesian optimisation. Median number of features across folds and range is reported.

| **Positive class** | **Hyperparameters** | **Features (score/ocurrence)** |
| --- | --- | --- |
| Atypical | Signature size: 2 [1,6] | Szm_glnu (56.4/67.6%)  Morph_vol_dens_aabb (54.8/56.5%) |
| Basal | Signature size: 3 [1,6] | Log_stat_mean (45.2/50.5%)  Log_ivh_i50 (39.0/38.3%)  Morph_vol_dens_aabb (34.5/40.4%) |
| Classical | Signature size: 2 [1,6] | Cm_joint_max_d1 (42.4/50.5%)  Morph_com (37.6/45.4%) |
| Mesenchymal | Signature size: 3 [1,6] | Morph_pca_elongation (59.1/59.9%)  Dzm_lde (54.8/63.6%)  Cm_joint_max_d1 (33.1/41.4%) |

**Supplementary Table 6:** Cutoffs for the output probabilities of the four subtype surrogate models alongside the atypical model on the HPV negative population. Output probabilities higher than the cutoff are labeled as the negative class. Cutoffs are chosen based on 600 bootstraps of the discovery cohort and chosen based on the cutoff with median f1 score higher than the reference f1 score of the discovery cohort (Supplementary Table 15) and with highest lower end of the 95% bootstraps CI.

| **Positive class** | **Cutoff** |
| --- | --- |
| Atypical | 0.161 |
| Basal | 0.353 |
| Classical | 0.080 |
| Mesenchymal | 0.131 |
| Atypical (HPV-) | 0.101 |

**Supplementary Figure 1:** Calibration plots for the atypical model in the discovery and validation cohorts for the atypical model including and excluding HPV+ patients. Slopes and inetrcepts with 95% CI are given alongside the p-value for the HL test.

**
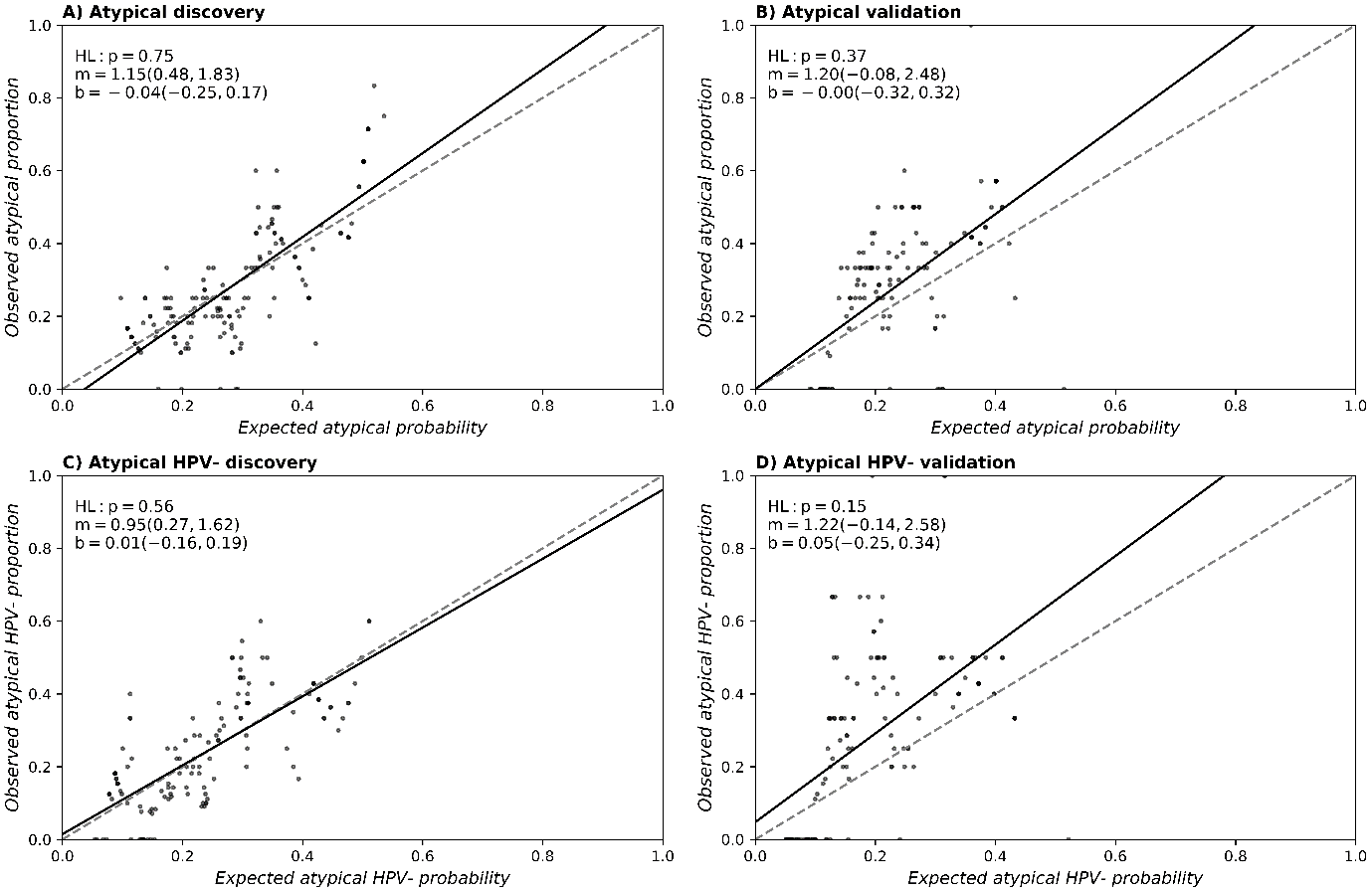
**

| Gene signature | Hyperparameters | Features (score/ocurrence) |
| --- | --- | --- |
| DNA repair | Signature size: 2 [1,5] | Cm_inv_var_d1 (34.8/49.4%)  Log_stat_range (31.4/49.4%) |
| Radiosensitivity | Signature size: 4 [1,6] | Cm_diff_avg (24.8/46.4%)  Cm_inv_var_d1 (21.3/34.3%)  Cm_info_corr2 (19.6/35.3%)  Morph_sph_dispr (15.4/31.3%) |
| Hypoxia | Signature size: 3 [1,5] | Rlm_rl_entr_3d_avg (41.9/58.5%)  Szm_glnu (39.7/57.5%)  Log_stat_min (20.3/31.3%) |
| Immunity | Signature size: 2 [1,5] | Log_stat_p90 (37.4/56.5%)  Morph_vol_approx (31.9/52.5%) |
| Radiosensitivity  (NCI 60) | Signature size: 2 [1,5] | Log_ivh_v50 (47.1/65.6%)  Stat_skew (33.3/49.4%) |
| EMT | Signature size: 4 [1,6] | Rlm_entr_3d (68.9/81.8%)  Log_stat_p90 (14.6/30.3%)  Morph_moran_i (14.2/28.2%)  Rlm_srhge_3d (11.05/26.26%) |

**Supplementary Table 7:** Selected features with enhanced Borda scores and occurrences across CV folds of the discovery cohort for each gene-signature model. All features were selected using the MRMR selection algorithm. The only hyperparameter was the number of selected features, which was optimized through Bayesian optimization. Median number of features across folds is reported

**Supplementary Table 8:** Performance of the radiomics logistic regression models to predict gene signature classification: median area under the curve (AUC) and accuracy (Acc) in discovery (disc) and validation (val) cohorts with 95% confidence intervals (CI) and p-value for the calibration using the Hosmer-Lemeshow (HL) test in validation.

| **Gene Signature** | **AUC disc [95% CI]** | **AUC val [95% CI]** | **Acc disc [95% CI]** | **Acc val [95% CI]** | **HL val p-value** |
| --- | --- | --- | --- | --- | --- |
| **DNA repair** | 0.61 [0.50-0.70] | 0.57 [0.46-0.68] | 0.61 [0.51-0.68] | 0.61 [0.51-0.71] | 0.094 |
| **Radiosensitivity** | 0.55 [0.44-0.66] | 0.33 [0.20-0.45] | 0.53 [0.50-0.58] | 0.52 [0.50-0.55] | 0.043 |
| **Immune processes** | 0.56 [0.45-0.66] | 0.52 [0.39-0.65] | 0.51 [0.47-0.56] | 0.57 [0.50-0.65] | 0.30 |
| **Hypoxia** | 0.68 [0.58-0.77] | 0.55 [0.43-0.68] | 0.65 [0.55-0.72] | 0.56 [0.47-0.65] | 0.042 |
| **Radiosensitivity (NCI 60)** | 0.58 [0.49-0.67] | 0.48 [0.35-0.61] | 0.57 [0.48-0.65] | 0.55 [0.45-0.64] | 0.032 |
| **EMT** | 0.67 [0.57-0.77] | 0.55 [0.42-0.67] | 0.63 [0.55-0.69] | 0.53 [0.46-0.61] | 0.23 |

*Abbreviations: EMT: Epithelial-mesenchymal transition*

**Supplementary Figure 2:** Boxplots of absolute Spearman correlations$\left\lfloor\rho\right\rfloor$ between individual radiomics features and genes of the six selected gene signatures in the discovery cohort, grouped by gene signature. No gene signature contained a gene with a correlation $\left\lfloor\rho\right\rfloor\geq0.45$ with a radiomic feature, indicating weak to moderate correlations between individual genes and radiomics features.

**
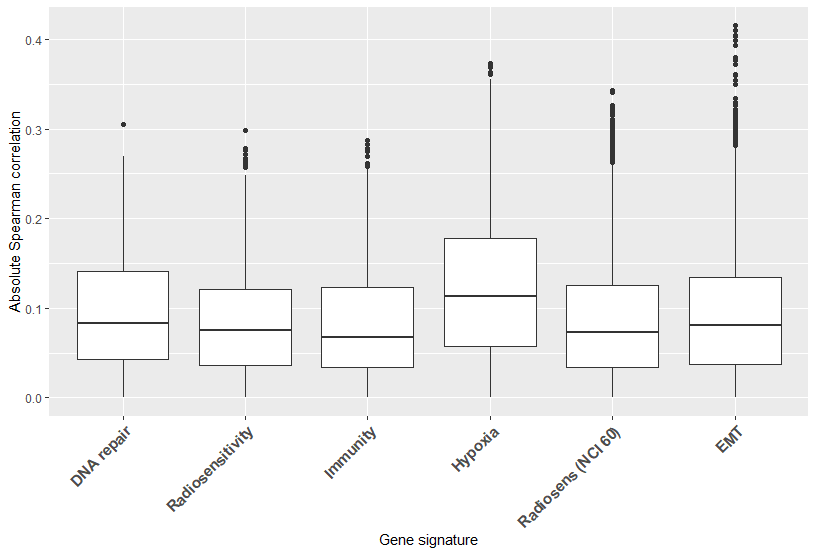
**

| **Model** | **Hyperparameters** | **Features (score/occurrence)** |
| --- | --- | --- |
| Radiomics | Signature size: 2 [2,4] | Morph_volume (50.58/100%)  Log_stat_p90 (8.55/31.3%) |
| Metagene | Signature size: 2 [1,3] | E2F_targets (17.18/42.4%)  Hedgehog_signaling (3.10/19.1%) |

**Supplementary Table 9:** Selected features with enhanced Borda scores and occurrences across CV folds of the discovery cohort for the radiomics and metagene models. All features were selected using the maximum-relevance minimum-redundancy (MRMR) selection algorithm. The only hyperparameter was the number of selected features, which was optimized through Bayesian optimisation. Median number of features across folds and range is reported. Volume had a 100% occurrence as it was always added to the feature selection process.

**Supplementary Figure 3:** Stratification (A, B, C) and calibration (D, E, F) of the radiomics (left), metagene (centre), and combined models (right) for the prognosis of loco-regional control (LRC) in the discovery cohort. Both metagene and radiomics achieved good calibration and stratification. When combining the radiomics signature and metagenes, a well-calibrated model with a more pronounced stratification in the validation cohort was obtained compared to the other two models.

**
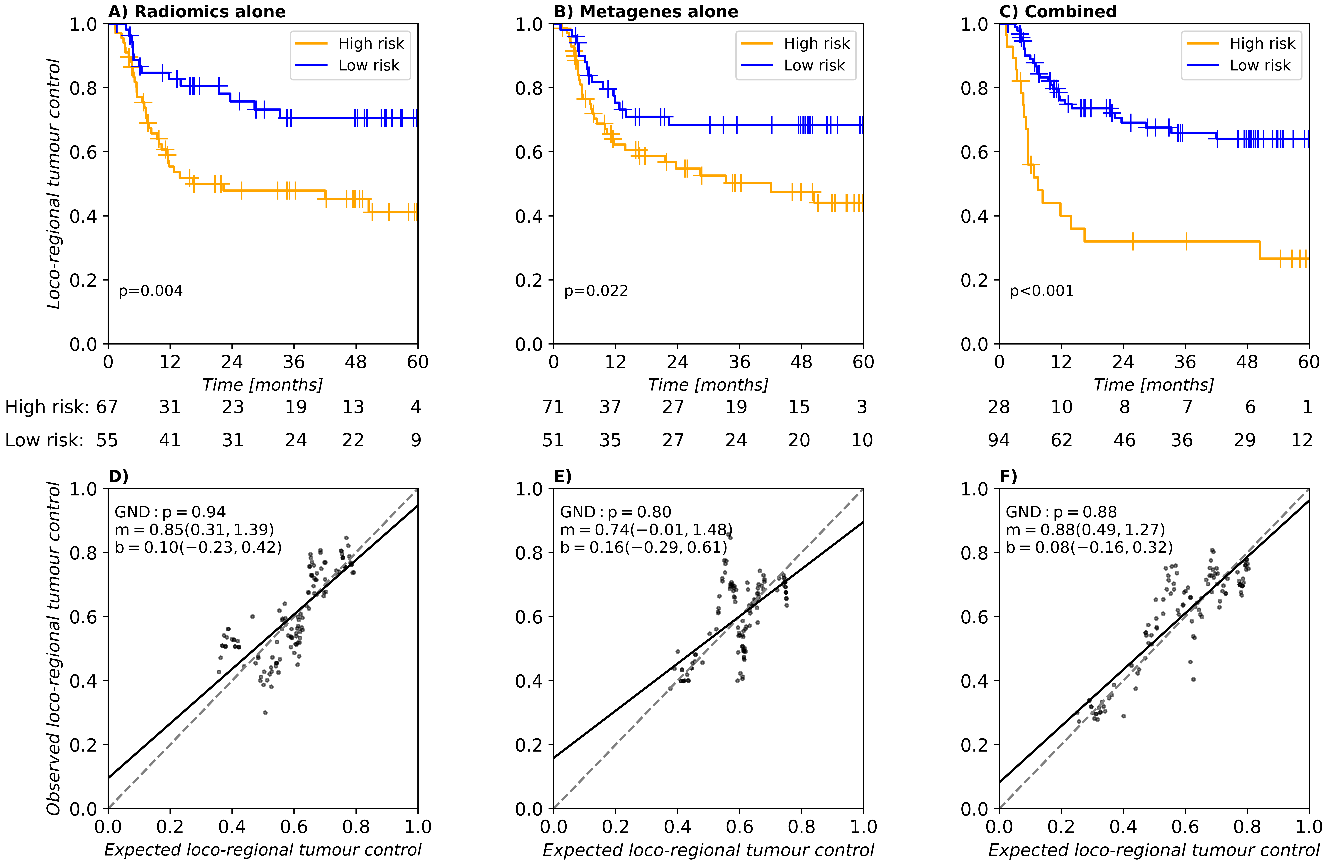
**

.

| *SHH* | *PTCH1* | *NRCAM* | *NRP1* | *SCG2* | *AMOT* | *UNC5C* |
| --- | --- | --- | --- | --- | --- | --- |
| *ADGRG1* | *HEY1* | *GLI1* | *THY1* | *SLIt1* | *CDK6* | *HEY2* |
| *NRP2* | *TLE3* | *TLE1* | *L1CAM* | *PLG* | *NF1* | *RASA1* |
| *ETS2* | *RTN2* | *CRMP1* | *MYH9* | *VEGFA* | *CELSR1* | *CNTFR* |
| *ACHE* | *PML* | *CDK5R1* | *VLDLR* | *OPHN1* | *LDB1* | *DPYSL2* |

**Supplementary Table 10:** Genes present in the hedgehog signaling metagene, specified in Gene Symbols.

**Supplementary Table 11:** Genes present in the E2F transcription network metagene, specified in Gene Symbols.

| *AK2* | *ANP32E* | *ASF1A* | *ASF1B* | | *ATAD2* | *AURKA* | *AURKB* | *BARD1* | *BIRC5* | *BRCA1* |
| --- | --- | --- | --- | --- | --- | --- | --- | --- | --- | --- |
| *BRCA2* | *BRMS1L* | *BUB1B* | *CBX5* | | *CCNB2* | *CCNE1* | *CCP110* | *CDC20* | *CDC25A* | *CDC25B* |
| *CDCA3* | *CDCA8* | *CDK1* | *CDK4* | | *CDKN1A* | *CDKN1B* | *CDKN2A* | *CDKN2C* | *CDKN3* | *CENPE* |
| *CENPM* | *CHEK1* | *CHEK2* | *CIT* | | *CKS1B* | *CKS2* | *CNOT9* | *CSE1L* | *CTCF* | *CTPS1* |
| *DCK* | *DCLRE1B* | *DCTPP1* | *DDX39A* | | *DEK* | *DEPDC1* | *DIAPH3* | *DLGAP5* | *DNMT1* | *DONSON* |
| *DSCC1* | *DUT* | *E2F8* | *EED* | | *EIF2S1* | *ESPL1* | *EXOSC8* | *EZH2* | *GINS1* | *GINS3* |
| *GINS4* | *GSPT1* | *H2AX* | *H2AZ1* | | *HELLS* | *HMGA1* | *HMGB2* | *HMGB3* | *HMMR* | *HNRNPD* |
| *HUS1* | *ILF3* | *ING3* | *IPO7* | | *JPT1* | *KIF18B* | *KIF22* | *KIF2C* | *KIF4A* | *KPNA2* |
| *LBR* | *LIG1* | *LMNB1* | *LUC7L3* | | *LYAR* | *MAD2L1* | *MCM2* | *MCM3* | *MCM4* | *MCM5* |
| *MCM6* | *MCM7* | *MELK* | *MKI67* | | *MLH1* | *MMS22L* | *MRE11* | *MSH2* | *MTHFD2* | *MXD3* |
| *MYBL2* | *MYC* | *NAA38* | *NAP1L1* | | *NASP* | *NBN* | *NCAPD2* | *NME1* | *NOLC1* | *NOP56* |
| *NUDT21* | *NUP107* | *NUP153* | *NUP205* | | *ORC2* | *ORC6* | *PA2G4* | *PAICS* | *PAN2* | *PCNA* |
| *PDS5B* | *PHF5A* | *PLK1* | *PLK4* | | *PM2* | *PNN* | *POLA2* | *POLD1* | *POLD2* | *POLD3* |
| *POLE* | *POLE4* | *POP7* | *PPM1D* | | *PPP1R8* | *PRDX4* | *PRIM2* | *PRKDC* | *PRPS1* | *PSIP1* |
| *PSMC3IP* | *PTTG1* | *RACGAP1* | *RAD1* | | *RAD21* | *RAD50* | *RAD51AP1* | *RAD51C* | *RAN* | *RANBP1* |
| *RBBP7* | *RFC1* | *RFC2* | *RFC3* | *RNASEH2A* | | *RPA1* | *RPA2* | *RPA3* | *RRM2* | *SHMT1* |
| *SLBP* | *SMC1A* | *SMC3* | *SMC4* | | *SMC6* | *SNRPB* | *SPAG5* | *SPC24* | *SPC25* | *SRSF1* |
| *SRSF2* | *SSRP1* | *STAG1* | *STMN1* | | *SUV39H1* | *SYNCRIP* | *TACC3* | *TBRG4* | *TCF19* | *TFRC* |
| *TIMELESS* | *TIPIN* | *TK1* | *TMPO* | | *TOP2A* | *TP53* | *TRA2B* | *TRIP13* | *TUBB* | *TUBG1* |
| *UBE2S* | *UBE2T* | *UBR7* | *UNG* | | *USP1* | *WDR90* | *WEE1* | *XPO1* | *XRCC6* | *ZW10* |

**Supplementary Figure 4**: Heatmap of the normalised feature expressions between high and low risk patients stratified by the optimised cutoff for the model combining metagenes and the radiomics signature. High risk patients tended to have a larger gross tumour volume (GTV), higher 90^th^ percentile on the Laplacian of Gaussian intensity histogram within the GTV (log_stat_p90) and hedgehog signalling metagene, while usually showing lower values of the E2F transcriptional targets metagene.


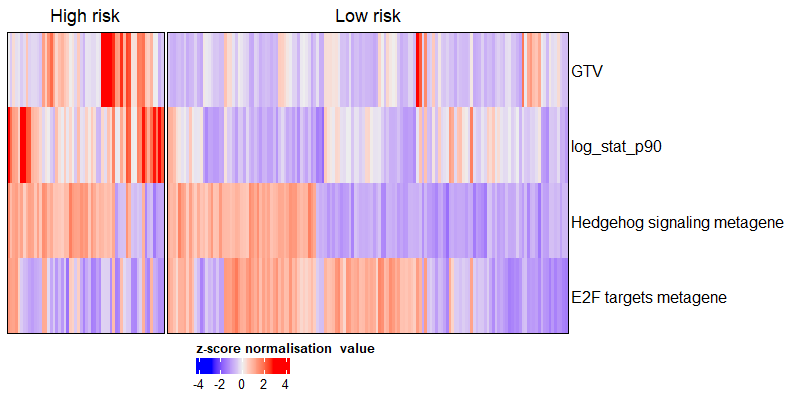


**Supplementary Table 12:** p-values of the $\chi^{2}$ test of the Schoenfeld residuals for each feature in each Cox model (radiomics alone, metagenes alone, and combined) for the signature combination study in the discovery cohort to justify the proportional hazards (PH) assumption. Non-significant p-values mean that the proportional hazards assumption holds.

| **Model** | **Feature** | **p-value** |
| --- | --- | --- |
| **Radiomics** | Morph_volume | 0.88 |
|  | Log_stat_p90 | 0.15 |
| **Metagene** | Hedgehog_signaling | 0.84 |
|  | E2F targets | 0.63 |
| **Combined** | Morph_volume | 0.90 |
|  | Log_stat_p90 | 0.18 |
|  | Hedgehog_signaling | 0.86 |
|  | E2F signaling | 0.71 |

**Supplementary Figure 5:** Kaplan-Meier and calibration plots for the GTV+metagenes model within the discovery (A and C) and validation (B and D) cohorts.


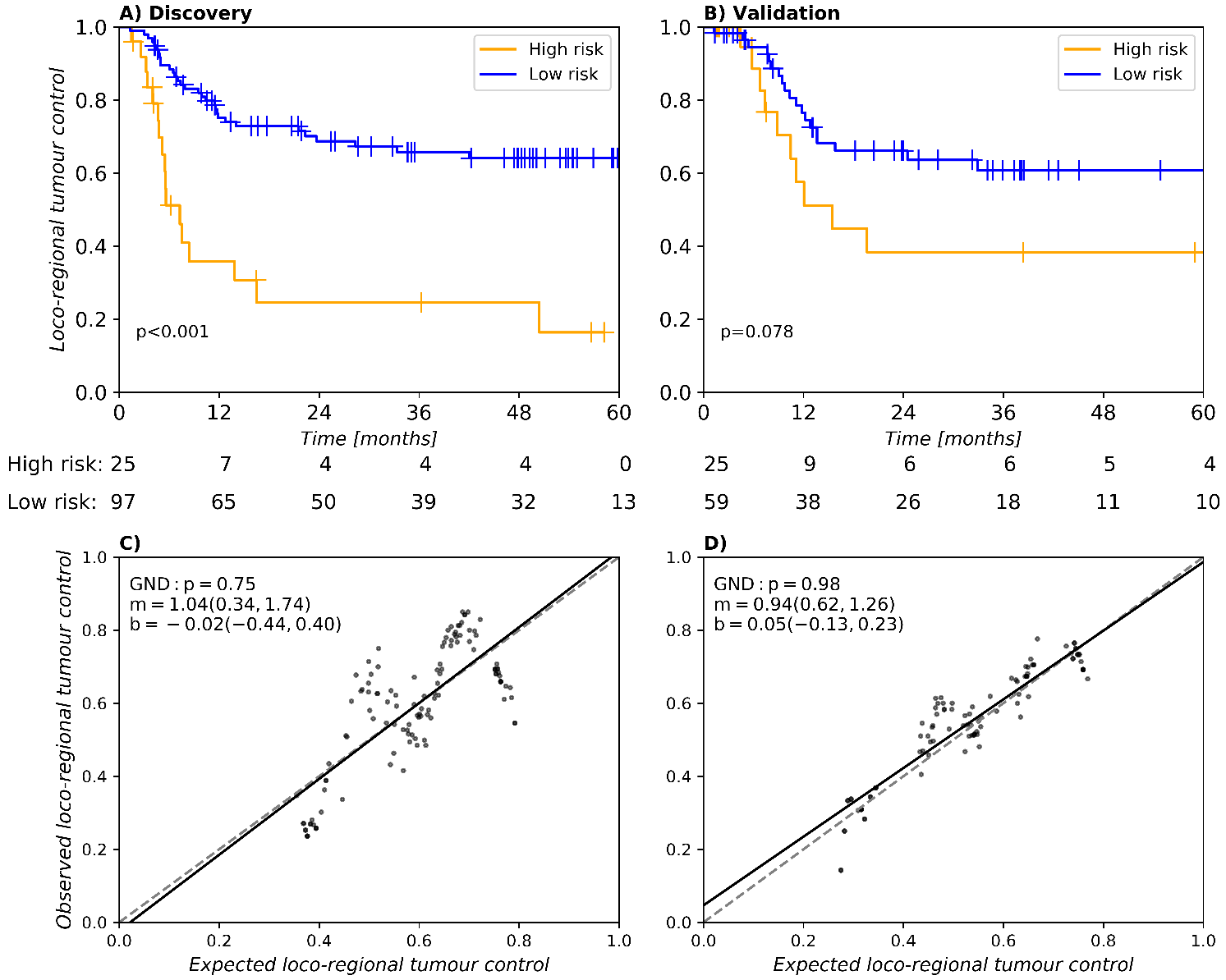


**Supplementary Table 13:** Scanner metadata for the discovery and validation cohort.

| **Image Acquisition Parameters** |  | **Discovery cohort (n=122)** | **Validation cohort (n=84)** | | |
| --- | --- | --- | --- | --- | --- |
| **Voxel spacing (x,y) in mm** | (0.85,0.85) | 2 |  | 0 |  |
|  | (0.87,0.87) | 2 |  | 0 |  |
|  | (0.88,0.88) | 1 |  | 0 |  |
|  | (0.90,0.90) | 1 |  | 0 |  |
|  | (0.92,0.92) | 1 |  | 0 |  |
|  | (0.94,0.94) | 2 |  | 0 |  |
|  | (0.95,0.95) | 2 |  | 0 |  |
|  | (0.96,0.96) | 3 |  | 0 |  |
|  | (0.97,0.97) | 5 |  | 0 |  |
|  | (0.98,0.98) | 49 |  | 50 |  |
|  | (1.17,1.17) | 13 |  | 0 |  |
|  | (1.27,1.27) | 20 |  | 0 |  |
|  | (1.37,1.37) | 21 |  | 34 |  |
| **Slice spacing (z) in mm** | 2 | 19 |  | 0 |  |
|  | 2.5 | 20 |  | 0 |  |
|  | 3 | 47 |  | 35 |  |
|  | 3.75 | 1 |  | 0 |  |
|  | 5 | 35 |  | 49 |  |
| **Reconstruction kernel** | B | 13 |  | 0 |  |
|  | B10s  B20f | 0  3 |  | 1  28 |  |
|  | B30f | 1 |  | 0 |  |
|  | B30s | 22 |  | 0 |  |
|  | B31f | 0 |  | 26 |  |
|  | B31s | 19 |  | 0 |  |
|  | B40s | 1 |  | 0 |  |
|  | STANDARD  B50s | 21  0 |  | 0  6 |  |
|  | 59.10.AB50  H31s | 15  2 |  | 0  0 |  |
|  | Missing | 25 |  | 16 |  |
| **Mean Exposure mA** |  | 262.16 (Missing: 27) |  | 60.05 (Missing:44) |  |
| **Manufacturer** | Siemens | 60 |  | 65 |  |
|  | MDS Nordion | 19 |  | 19 |  |
|  | GE Medical Systems | 26 |  | 0 |  |
|  | Picker International | 2 |  | 0 |  |
|  | Philips | 15 |  | 0 |  |
| **Scanner Model** | Biograph16 | 0 |  | 26 |  |
|  | Sensation16  Emotion | 3  0 |  | 29  6 |  |
|  | Somatom PLUS4 | 15 |  | 0 |  |
|  | Sensation Open | 42 |  | 0 |  |
|  | Lightspeed Ultra | 21 |  | 0 |  |
|  | PQ5000 | 2 |  | 0 |  |
|  | Brilliance Big Bore | 13 |  | 0 |  |
|  | Volume Zoom | 1 |  | 0 |  |
|  | Missing | 25 |  | 23 |  |
| **Mean exposure time (ms)** | 500  719  750  800  806  1000  Missing | 4  20  0  0  1  57  40 |  | 54  0  1  6  0  0  23 |  |
| **Tube voltage in kV** | 120 | 80 |  | 55 |  |
|  | 130 | 2 |  | 6 |  |
|  | 140 | 15 |  | 0 |  |
|  | Missing | 25 |  | 23 |  |

**Supplementary Table 14:** Table with specifications and algorithms used for extraction of radiomic features from the pre-treatment CT data.

| **Image interpolation** | |
| --- | --- |
| Interpolation method | Cubic spline |
| Voxel dimensions in mm3 | 1 x 1 x 1 |
| Smoothing parameter β | 0.98 |
| **Windowing** |  |
| Hounsfield unit (HU) range (min, max) | -150, 180 |
| **ROI interpolation** | |
| Interpolation method | Cubic spline |
| Inclusion threshold | 0.5 |
| **Discretization** | |
| Discretization method | Fixed Bin Number (FBN) of 32 bins |
| Intensity Volume Histogram discretization method | Fixed Bin Number (FBN) of 1000 bins |
| **Image transformation** | |
| Image filter | Mean-Intensity Laplacian of Gaussian (1,2,3,4,5 mm) |
| **Texture matrices** | |
| Grey-level Run Length Matrix (GLRLM) | Calculation method: 3D  Merge method: volume merge |
| Grey-level Size Zone Matrix (GLSZM) | Calculation method: 3D |
| Neighbourhood Grey Tone Difference Matrix (NGTDM) | Calculation method: 3D |
| Neighbourhood Grey Level Dependence Matrix (NGLDM) | Distance for neighborhood: 1.8 voxels  Difference level: 0.0  Calculation method: 3D |
| Grey Level Co-occurrence Matrix (GLCM) | Distance for neighborhood: 1.0 voxels  Calculation method: 3D  Merge method: volume merge |
| Grey Level Distance Zone Matrix (GLDZM) | Calculation method: 3D |

**Supplementary Table 15:** Parameters used for consensus clustering through the ConsensusClusterPlus function for the radiomics intensity, texture, and statistical features.

| **Parameter** | **Value** |
| --- | --- |
| seed | 42 |
| reps | 2000 |
| clusterAlg | hc |
| distance | euclidean |
| pItem | 0.75 |
| innerLinkage | average |
| finalLinkage | average |

Abreviations: Hc: hierarchical clustering, pItem: resampling percentage of samples (patients).

**Supplementary Figure 6:** Tracking plot of the cluster membership k (rows) for each patient (column). Patients that do not change colors when k increases remain within the same cluster. Many patients were observed to remain in the biggest cluster (light blue). The subset of data points that always remained in that cluster regardless of the value of k was taken as the reference batch.


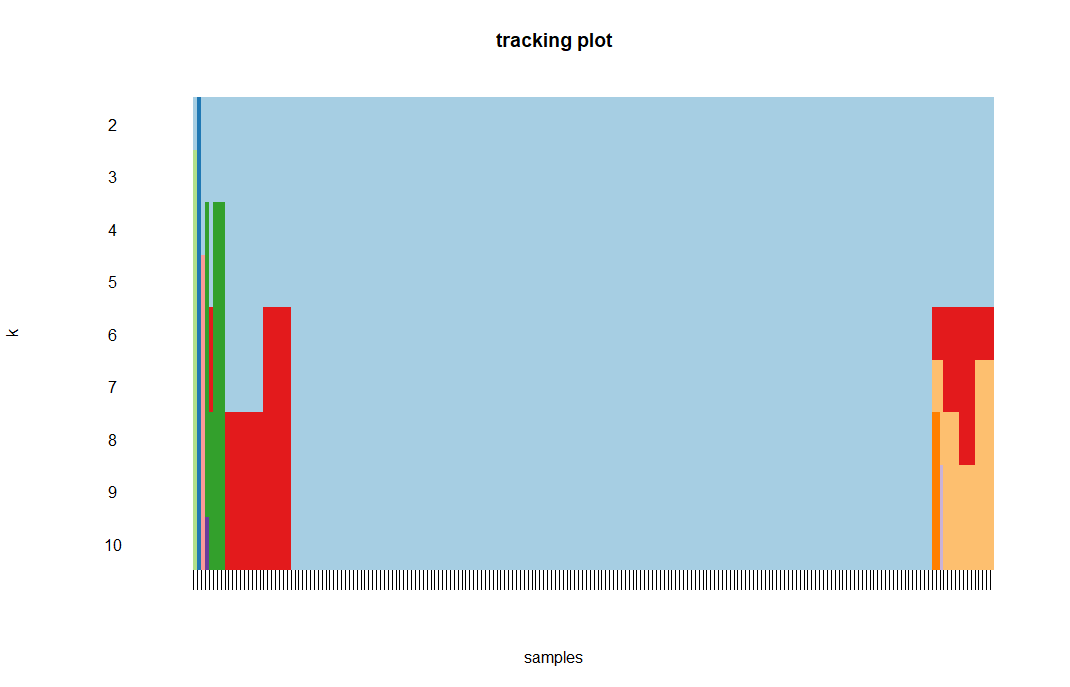


**Supplementary Figure 7:** 3D PCA plot with percentage of explained variance per axis, of the radiomics features related to statistical, intensity, and texture of the tumours, with each dot representing a patient. Patients are grouped in two clusters based on consensus clustering results. All points not belonging to the largest, most conserved cluster (Reference) were taken as outliers (Not reference) and subsequently adjusted to the reference cluster through non-parametric ComBat normalisation. The more dispersed disposition of the non-reference data points can be observed qualitatively in the plot.


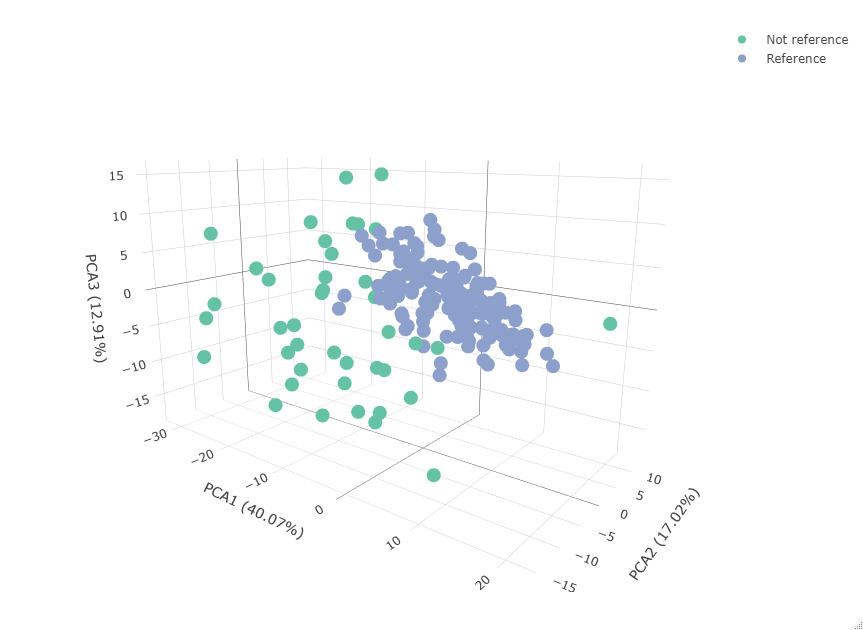


**Supplementary Table 16:** Parameters used for GSVA metagene calculation using the gsva function of the R package GSVA.

| **Parameter** | **Value** |
| --- | --- |
| method | gsva |
| kcdf | Gaussian |
| mx.diff | FALSE |

**Supplementary Table 17:** Pathways from the hallmarks of cancer dataset from MSigDB that remained after bibliographical search for potential association with HNSCC.

| Pathway |
| --- |
| Hallmark_TNFA_signaling_via_NFKb |
| Hallmark_hypoxia |
| Hallmark_cholesterol_homeostasis |
| Hallmark_WNT_beta_catenin_signaling |
| Hallmark_IL6_STAT3_signaling |
| Hallmark_DNA_repair |
| Hallmark_apoptosis |
| Hallmark_notch_signaling |
| Hallmark_estrogen_response_early |
| Hallmark_estrogen_response_late |
| Hallmark_hedgehog_signaling |
| Hallmark_unfolded_prot_response |
| Hallmark_PI3K_AKT_MTOR_signaling |
| Hallmark_MTORC1_signaling |
| Hallmark_E2F_targets |
| Hallmark_MYC_targets_v1 |
| Hallmark_MYC_targets_v2 |
| Hallmark_EMT |
| Hallmark_inflammatory_response |
| Hallmark_fatty_acid_metabolism |

**Supplementary Section: Reference values for f1 scores**

To better understand the performance of the classification models for subtype classification, we give reference values for the f1 scores, as a value of 0.5 is not indicative per se of an uninformative model due to the f1 score being a harmonic mean of two other scores: precision and recall.

Reference values for the f1 score depend on the proportion of the positive class and can be derived considering a trivial model with binary classes that always picks the positive class. If q is the positive class proportion, the f1 score of this model would be $f_{1,\mathrm{trivial}}=\frac{2q}{q+1}$.

**Supplementary Table 18**: Reference values for the f1 scores for each subtype and cohort. An f1 score above the reference value indicates better performance than the “best” trivial model, one that always selects the positive class.

| Positive class | Cohort | Positive fraction (%) | f1 reference |
| --- | --- | --- | --- |
| Atypical | Discovery | 29.0 | 0.45 |
| Atypical | Validation | 29.3 | 0.45 |
| Basal | Discovery | 33.3 | 0.50 |
| Basal | Validation | 36.6 | 0.54 |
| Classical | Discovery | 17.4 | 0.29 |
| Classical | Validation | 12.2 | 0.21 |
| Mesenchymal | Discovery | 20.3 | 0.34 |
| Mesenchymal | Validation | 21.9 | 0.36 |
| Atypical (HPV-) | Discovery | 23.8 | 0.38 |
| Atypical (HPV-) | Validation | 27.0 | 0.43 |
